# Quality Information about Uterine Fibroids on the Internet

**DOI:** 10.1055/s-0038-1672163

**Published:** 2018-09

**Authors:** Daniela Gama de Melo, Pedro Sérgio Soares Jallad, Luiz Gustavo Oliveira Brito

**Affiliations:** 1 Department of Gynecology and Obstetrics, Faculty of medicine of Ribeirão Preto, Universidade de São Paulo, Ribeirão Preto, SP, Brazil; 2 Department of Gynecology and Obstetrics, Faculty of Medical Sciences, Universidade Estadual de Campinas, Campinas, SP, Brazil

**Keywords:** uterine fibroids, quality of information, DISCERN questionnaire, internet, miomas uterinos, qualidade da informação, questionário DISCERN, internet

## Abstract

**Objective** There are no published studies analyzing the quality of the information for lay women on the Internet regarding uterine fibroids. The accuracy of the provided material is also unknown. Thus, we have performed a cross-sectional study with 381 websites in the English and Brazilian Portuguese languages between May and December 2017.

**Methods** Two investigators performed the analysis, and the Cohen kappa coefficient was calculated to analyze the agreement between them. Search terms (*uterine fibroids* and derivatives) in the English and Brazilian Portuguese languages were used. The accuracy was analyzed by a 10-item checklist created based on the American Society for Reproductive Medicine (ASRM), National Institutes of Health (NIH) and European Menopause and Andropause Society (EMAS) consensuses about uterine fibroids. The item–test correlation and the intraclass coefficient were performed in the 16 questions from the DISCERN instrument, which was designed to measure the quality of health information on the Internet. Analysis of variance (ANOVA) measurements were performed for the independent variables and the DISCERN/accuracy scores.

**Results** Google was the most used search engine, and uterine fibroid was the search term that generated most of the analyzed material. The median score for accuracy in all websites was 5 out of 10, and the median score of the DISCERN instrument was 38 out of 80. The top-scoring sites in the English language were derived from scientific organizations and federal governments, and they regarded the DISCERN score (The American College of Obstetricians and Gynecologists [ACOG], the Food and Drug Administration [FDA]) and the accuracy criteria (NIH, and FDA). On the other hand, in the Brazilian Portuguese language, the highest scores in both instruments were from magazines or physician's blogs. The Cronbach α test showed a higher correlation (0.77–0.79) between the sites and DISCERN; however, the item–test correlation varied from 0.39 to 0.56.

**Conclusion** There is a need to improve the quality of the information regarding uterine fibroids for lay women.

## Abstract (pt)

**Resumo:**

**Objetivos** Não existem estudos publicados analisando a qualidade de informação para mulheres leigas na internet sobre o leiomioma uterino. A acurácia do material existente também não é conhecida. Portanto, realizamos um estudo transversal envolvendo 381 sites em língua inglesa e portuguesa de maio a dezembro de 2017.

**Métodos** Dois pesquisadores fizeram a análise, e o coeficiente kappa de Cohen foi calculado para analisar a concordância entre ambos. Termos de pesquisa (*leiomioma uterino* e derivados) em inglês e português foram usados. A acurácia foi analisada por meio de uma lista de 10 itens criados após a fusão de consensos da Sociedade Americana de Medicina da Reprodução (ASRM, na sigla em inglês), dos Institutos Nacionais de Saúde (NIH, na sigla em inglês) e da Sociedade Europeia de Menopausa e Andropausa (EMAS, na sigla em inglês) sobre leiomioma uterino. A correlação item–teste e o coeficiente intraclasse foram realizados nas 16 questões do questionário DISCERN, um instrumento desenvolvido para medir a qualidade da informação de saúde disponível na internet. O método de análise de variância (ANOVA, na sigla em inglês) foi utilizado para as variáveis independentes e as pontuações de acurácia e do DISCERN.

**Resultados** O Google foi a ferramenta mais utilizada, e o leiomioma uterino foi o termo de busca que gerou a maior parte do material analisado. A pontuação média para a acurácia dos websites foi 5/10, e do questionário DISCERN, 38/80. Os sites de língua inglesa com maior pontuação foram os de organizações científicas e de governos federais, tanto no questionário DISCERN (Faculdade Americana de Obstetrícia e Ginecologia [ACOG, na sigla em inglês], Administração de Alimentos e Medicamentos [FDA, na sigla em inglês]) quanto na acurácia (NIH e FDA). Entretanto, em língua portuguesa, os sites com as maiores pontuações em ambos os instrumentos foram de revistas ou blogs médicos. O teste α de Cronbach evidenciou maior correlação entre os sites e o DISCERN (0,77–0,79); contudo, a correlação item–teste variou de 0,39 a 0,56.

**Conclusão** Há necessidade de melhorar a qualidade da informação sobre o leiomioma uterino para mulheres leigas.

## Introduction

Uterine fibroids (UFs) are the most common benign gynecological pathology, and out of the 70 to 80% of women who present these tumors, 20 to 30% will have symptoms related to their presence, such as abnormal uterine bleeding and pelvic pain.^1^ These complaints significantly impair their quality of life,^2^ and UFs are the main reason for benign hysterectomy in the United States.^3^ Despite the advance of non-surgical treatment for UFs, we still do not have a non-invasive option with no side effects that may enable the avoidance hysterectomy and/or myomectomy in all cases.

Furthermore, one of the motivations for women to undergo surgery is the lack of knowledge regarding the disease.^4^ The Internet is an option for seeking information, and the number of users has considerably increased year after year; however, the quality of the medical information available for lay women is extremely variable.^5^ The number of websites presenting topics directly and/or indirectly related (with mention, for example) to UFs has risen from 5,680 to 70,600 since 2004, and the absolute number of searches has increased. However, the mean activity of global search for UF has reduced, which may represent a higher availability of material regarding the disease, or websites with low quality of information.^6^

To our knowledge, there are scant data regarding the quality of the information available about UF. There are tools available to assess the quality of health-related information, such as the DISCERN instrument,^7^ which has been extensively used in the medical literature to assess these points. Moreover, the accuracy of the content is another issue that should be assessed while reading these materials. Given that, we have aimed to evaluate the quality of the information regarding UF on the Internet by searching patient-focused, professional, governmental and consumer websites.

## Methods

### Study Design, Identification of the Websites

The present review was registered at the PROSPERO database (CRD42015017139). The study protocol was presented to our Institutional Review Board, which authorized the study. A list of terms derived from uterine fibroids (uterine leiomyoma, fibroids, myoma, for example) in the English and Brazilian Portuguese languages were investigated on several search engines (Google, Bing, Yahoo). Websites that were not available for patient information or with scientific studies with no plain summary were excluded. We have also actively sought for patient-information websites contained within the most important scientific associations related to gynecology (such as the American Society for Reproductive Medicine [ASRM] and the European Society of Human Reproduction and Embryology [ESHRE]), or websites belonging to the federal government (such as those of the National Institutes of Health [NIH] and the Food and Drug Administration [FDA]) in both languages. Two independent investigators (Melo DG, first-year resident of obstetrics and gynecology, and Jallad PSS, a medical intern), performed this search with no discussion between them. A third investigator, Brito LGO, an experienced physician, double-checked the first 30 scores from both languages to see if there was any disagreement.

### Instruments

The assessment of the accuracy of the content consisted of a compilation of all definition, diagnosis, and treatment consensuses of the ASRM, the NIH, and the European Menopause and Andropause Society (EMAS).^8^ ^9^ Each item received a score (0–incorrectly mentioned or not mentioned; 1–partially or totally/correctly mentioned), with a possible range between 0 and 10, which is displayed in Table 1.

**Table 1:** Accuracy criteria for the information regarding uterine fibroids found in websites

Item	Description
1	Definition – a gynecological benign tumor, with low risk potential for cancer, affecting 20–30% of women, with symptoms such as abnormal bleeding and pelvic pain
2	Risk factors – African American women, family history, obesity, hypertension
3	Clinical evaluation – anamnesis + physical exam findings, other symptoms, other aspects (e.g., fibroids and infertility)
4	Imaging evaluation – ultrasound and magnetic resonance imaging
5	Treatment options – expectant, clinical, minimally invasive, and surgical
6	Clinical treatment (hormonal) – progestogens, combined contraceptives, GnRH analogues, SPRMs
7	Clinical treatment (non-hormonal) – NSAIDs, antifibrinolytics
8	Minimally invasive treatment – uterine artery embolization, high intensity focused ultrasound
9	Surgical treatment – indications, myomectomy, hysterectomy
10	Decision-making process, morcellation and risk of incidental leiomyosarcoma

Abbreviations: GnRH, gonadotropin-releasing hormone; NSAID, non-steroidal anti-inflammatory drug; SPRM selective progesterone receptor modulator.

The quality of the information was analyzed through the DISCERN instrument, which is a 16-item questionnaire, with each question scored using an ordinal Likert scale (scores: 1–5), in which 5 indicates the highest quality. The individual scores are added, for a maximum attainable score of 80, and the highest scores are associated with higher quality and reliable information.^7^ We have also categorized the score results from the DISCERN questions into low (1), moderate (2–3), and high (4–5).

### Statistical Analysis

Data were collected using the Microsoft Excel 2013 (Microsoft Corporation, Redmond, WA, US) and exported to the Stata Statistical Software: Release13 (StataCorp LLC, College Station, TX, US) for statistical analysis. A descriptive analysis of each variable (median or mean, standard deviation [SD] or range, and interquartile values) was made. A Cohen kappa analysis was conducted, in which the two reviewers graded the scores in the DISCERN instrument and in the accuracy criteria. The intraclass coefficient and the item–test correlation were calculated for all 16 questions of the DISCERN instrument and of the accuracy criteria; a value lower than 0.60 indicated a low concordance; a score between 0.60 and 0.80 indicated a moderate concordance; and a score > 0.80 indicated a higher concordance. A linear regression was performed to compare the DISCERN instrument and the accuracy criteria. Analysis of variance (ANOVA) was used to compare any differences between the accuracy criteria or the DISCERN instrument among the search engines. The significance level was established at 5%.

## Results

After entering all of the search terms into the search engines, the results were retrieved within a range between 2 and 3.550.000 websites. We decided to search the first 200 results of each search engine. After excluding duplicates, of the 426 websites that were retrieved during the search, 380 were analyzed by both researchers.

Table 2 displays the main findings. Most of the pages did not have the year of publication. Google was the most used search engine, and *uterine fibroids* was the search term that generated most of the analyzed material. The ANOVA analysis showed a statistical difference regarding the search term and the DISCERN total score (*p* = 0.04), and no difference according to the accuracy criteria (*p* = 0.56). The most prevalent suffixes were *.com* (66.58%), and *.org* (18.42%), with no differences between them and the DISCERN questionnaire (*p* = 0.93). No statistical differences were observed regarding the search engine and the accuracy results (*p* = 0.31), but there were differences between them and the DISCERN instrument (*p* < 0.001).

**Table 2:** Descriptive features of the 380 reviewed websites

Variables	*n* (%)
Site suffix	
.com	253 (66.58%)
.edu	12 (3.16%)
.gov	9 (2.37%)
.org	70 (18.42%)
.info	2 (0.53%)
.net	23 (6.05%)
Other	11 (2.89%)
Search engine	
Google	180 (47.36%)
Yahoo	100 (26.32%)
Bing	100 (26.32%)
Search term (English/Brazilian Portuguese)	
Uterine fibroids/Fibroma uterino	150 (40.21%)
Uterine leiomyoma/Leiomioma uterino	98 (26.27%)
Uterine myoma/Mioma uterino	52 (13.94%)
Myoma/Mioma	73 (19.57%)
Year of publication/update (*n* = 373)	
Not informed/not found	336 (90.08%)
2015	1 (0.27%)
2016	1 (0.27%)
2017	35 (9.38%)
Site language	
English	305 (80.26%)
Brazilian Portuguese	75 (19.74%)
Total Internet site score for accuracy (median; IQR; range)	5; 3–7; 0–10
Total Internet site score for the DISCERN questionnaire (median; IQR; range)	38,35; 41.75; 16–72
Top-scoring Internet sites – English language (DISCERN score from 16 to 80)	
https://www.acog.org/-/media/For-Patients/faq074.pdf	72
https://www.fda.gov/downloads/AdvisoryCommittees/ CommitteesMeeting-Materials/MedicalDevices/MedicalDevicesAdvisoryCommittee/Obstetricsand-GynecologyDevices/UCM404865.pdf	71
https://www.mayoclinic.org/diseasesconditions/uterine-fibroids/diagnosistreatment/drc-20354294	71
https://www.womenshealth.gov/a-ztopics/uterine-fibroids	70
https://www.asrm.org/topics/topics-index/fibroids-or-myomas/	67
Top-scoring Internet sites – Brazilian Portuguese language (DISCERN score from 16 to 80)	
http://medsimples.com/mioma-uterino/	71
http://www.henriqueelkis.com.br/tipos_miomas.asp	69
http://www.portalsaofrancisco.com.br/saude/mioma	68
http://claudia.abril.com.br/saude/mioma-esclareca-14-duvidas-sobre-esse-tipo-de-tumor/	64
http://www.webmioma.com.br/	64
Top-scoring Internet sites – English language (Accuracy content from 0 to 20)	
https://www.nichd.nih.gov/health/topics/uterine/clinicaltrials/Pages/default.aspx	10
https://www.healthline.com/health/uterine-fibroids	10
https://www.mayoclinic.org/diseases-conditions/uterine-fibroids/diagnosis-treatment/drc-20354294	10
https://www.fda.gov/downloads/AdvisoryCommittees/CommitteesMeetingMaterials/MedicalDevices/MedicalDevicesAdvisoryCommittee/ObstetricsandGynecologyDevices/UCM404865.pdf	10
https://www.acog.org/-/media/For-Patients/faq074.pdf	9
Top-scoring Internet sites – Portuguese language (Accuracy content from 0 to 20)	
http://medsimples.com/mioma-uterino/	10
http://www.henriqueelkis.com.br/tipos_miomas.asp	10
http://www.portalsaofrancisco.com.br/saude/mioma	10
http://claudia.abril.com.br/saude/mioma-esclareca-14-duvidas-sobre-esse-tipo-de-tumor/	10
http://www.endoscopiaginecologica.med.br/index.php?option=com_k2&view=item&id=2:miomas&Itemid=176	10

Abbreviation: IQR, interquartile range.

The top-scoring sites in the English language were from scientific organizations and federal government agencies, and they regarded the DISCERN score (ACOG, ESHRE, and FDA) and the accuracy criteria (NIH, and FDA). The ASRM presented accuracy and DISCERN scores of 8 and 67 respectively. Moreover, we have found websites by general practitioners (American Academy of Family Physicians [AAFP]) explaining fibroids, with accuracy and DISCERN scores of, 7 and 64 respectively. On the other hand, in the Brazilian Portuguese language, the highest scores in both instruments were found in magazines or in physician's blogs (Table 2).

**Fig. 1:**
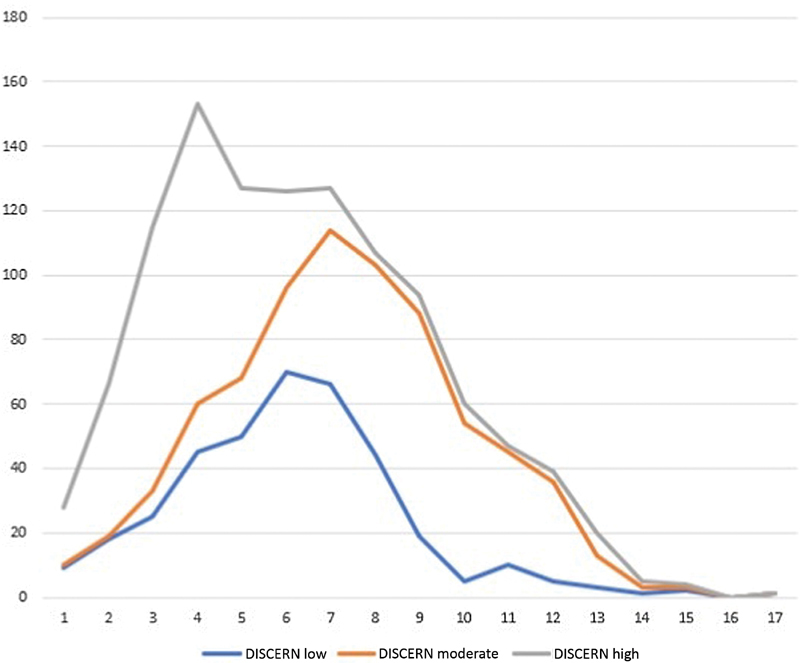
Distribution of DISCERN scores (low, moderate, high).

The median score for accuracy in all websites was 5 out of 10, and the median score of the DISCERN instrument was 38 out of 80. The linear regression showed a positive correlation between the accuracy criteria and the DISCERN instrument (exponent coefficient = 2.17 [1.54–3.04]; *p* < 0.001). The Cohen kappa results between the investigators regarding the accuracy and DISCERN analyses were, respectively, 0.78 and 0.72, showing a moderate agreement. Fig. 1 shows the distribution of the DISCERN answers by category; a broader, distributed number of low scores is perceived throughout the graphic, different from moderate and high scores, which suggests that a higher number of low scores were found.

Table 3 displays the intraclass coefficient and the item–test correlation for all the DISCERN questions. The Cronbach α test showed a narrow range (0.77–0.79) between them, indicating a good to excellent correlation. However, the item–test correlation varied between 0.39 and 0.56, and, interestingly, question 16 (which represents the overall rating score of the publication) showed the lowest value, indicating a mild agreement with the rest. The mean DISCERN score for question 16 was 2.58, that is, most of the websites fulfilled between 50 and 60% of the DISCERN scores.

**Table 3:** Descriptive results, item–test correlation and intraclass coefficient of the DISCERN instrument for uterine fibroids (*n* = 380)

Questions* (Likert score: 1–5)	Total DISCERN score – Mean (SD)	Item-test correlation	Intraclass coefficient
1. Is it clear what the website is about, or that it meant to cover uterine fibroids?	3.61 ± 0.89	0.47	0.78
2. Does the website achieve its aims of discussing the definition, diagnosis and treatment of uterine fibroids?	3.10 ± 1.07	0.53	0.78
3. Does the website address the questions women might ask? Does it suggest viable treatment options?	2.85 ± 1.22	0.57	0.77
4. Does the website mention its sources of information? Does it refer to where the reader may obtain information about what is written?	2.06 ± 1.29	0.48	0.78
5. Is it clear when the document/its sources were produced describing the treatment options for uterine fibroids?	1.42 ± 1.03	0.53	0.78
6. Is the publication balanced/unbiased in presenting all of the treatment options for uterine fibroids?	2.91 ± 0.95	0.42	0.79
7. Does it provide details of additional sources of support and information?	1.81 ± 0.94	0.52	0.78
8. Does the website provide any gaps of knowledge or differences in expert opinion concerning the treatment choices for uterine fibroids?	2.11 ± 0.91	0.50	0.78
9. Does it describe how each treatment works?	2.7 ± 1.40	0.53	0.78
10. Does it describe the benefits of each treatment?	2.52 ± 1.15	0.56	0.77
11. Does it describe the risks of each treatment?	2.19 ± 1.05	0.61	0.77
12. Does it describe what would happen if no treatment is chosen?	1.51 ± 0.97	0.61	0.77
13. Does it describe how the treatment choices affect the overall quality of life of women with uterine fibroids (e.g., side effects, duration of treatment)?	2.63 ± 1.35	0.43	0.78
14. Is it clear that there may be more than one possible treatment choice?	3.14 ± 1.60	0.40	0.79
15. Does the website give suggestions for shared decision-making (with family, friends and health professionals)?	1.92 ± 1.18	0.48	0.78
16. What is the overall quality of the publication as a source of information about treatment choices?	2.58 ± 1.35	0.39	0.79

Abbreviation: SD, standard deviation.

Note: *Section 1 (Is the publication reliable?): questions 1–8; Section 2 (How good is the quality of information on treatment choices?): questions 9–15; Section 3 (Whart is the overall rating of the publication?): question 16.

## Discussion

In summary, we have found that websites with patient-health information about uterine fibroids presented an approximate score between 40 and 50% after being assessed by a quality information instrument (DISCERN), and 50% of accuracy of the provided information. However, the ACOG, and FDA websites presented higher DISCERN and accuracy scores, and it seems that, in the English language, scientific organizations and federal government agencies are aiming to communicate in a way that enables lay women to understand this disease. This involvement is essential to the credibility and proximity of these institutions with our patients. Recently, the FDA did not recommend electromechanical morcellation during laparoscopic hysterectomy due to the risk of incidental leiomyosarcoma, and this raised the number of searches about the topic on the Internet for some time.^10^ Information should be evidence-based, understandable by patients, and free of any bias. Conversely, the Brazilian Portuguese sites that achieved the highest scores were those of magazines or of physicians bloggers. It is important that medical societies be involved in the discussion of high-quality information for the patients.

The median accuracy score was 5 out of 10, and the median DISCERN score was 38 out of 80, and the percentage of low Likert scores among the respondents was higher than the percentage of high Likert scores. Both presented a positive correlation after the linear regression was performed. Thus, these data suggest that we have many websites discussing UFs, but few of them present good quality of information for the patients. Other gynecological diseases, such as pelvic organ prolapse, showed similar results^11^ regarding incomplete web-based information. It is important to inform women where the best reliable sources of information are, and this justifies the rationale for performing these types of studies.

When analyzing the DISCERN scores, the intraclass coefficients were higher for all questions, revealing that the results were similar to those of the respondents for each question. However, the item -test correlation results were lower, suggesting that each question may not be measuring the same construct when compared to other questions. This should be seen with caution, because subjectivity plays a role in lowering these values, and we have previous studies showing that the DISCERN instruments may be heavily influenced by the opinion of the investigator, reducing the interaction between the constructs of the questionnaire.^11^ ^12^ Nevertheless, a study has shown that the evidence-based quality rating and average DISCERN ratings are similar for both consumers and health professionals.^13^

A total of 90% of the websites did not present the year of publication. Other studies have found the same results.^12^ It is already known that all information that is directly prepared for the patient should be thoroughly discussed before publication, and the content should be updated when any news arises regarding diagnosis and treatment.^14^

This is the first study analyzing the quality of information about UFs, a prevalent disease in gynecology. Recently, a systematic review was performed for health information online regarding endometriosis, another common disease, and the authors had the same difficulty in finding accurate information, with most of the data having low quality.^15^ We have also taken the care to specifically evaluate the professional and governmental websites that are important referral sources for this disease. Cohen kappa coefficients of 0.72 and 0.73 regarding these 2 criteria between our investigators suggest that the agreement rate was high and reduced the risks of disagreements. However, some limitations should be addressed. The inclusion of only English and Brazilian Portuguese languages may have excluded other websites with potential higher scores, or may have occulted a higher low rate of websites. There may be a selection bias, because not all of the retrieved results were read; a criterion was applied to select them. However, we actively sought for patient-health information websites that could contain reliable, high-quality material by looking at the scientific association pages. Furthermore, other studies did not analyze all the data on the Internet because there is a huge amount of data to be scanned. Another point is that the DISCERN instrument includes the subjectivity of the overall rating question, and does not assess certain additional quality indicators of patient-health information, such as the readability. There are some sites that are certified by Health on the Net (HON), but a previous study investigating other gynecological diseases found low DISCERN scores that were HON-certified.^12^

## Conclusion

The Internet is a tool that has been exhaustively used by patients to obtain information. This might have the impact of expediting the decision-making process concerning the treatment for UFs if women decide to consult the World Wide Web before a medical consultation. We know that the retrieved results from a specific term may differ geographically and due to financial reasons, and that accurate, good information may not be the first option available to these women when they are seeking for data regarding their disease and treatment options. We have also shown that, in the English language, most of the websites are from hospitals, scientific organizations or federal government agencies, and this may guide the providers to educate our patients. Future research is necessary, with the inclusion of more variables, such as readability, and with the participation of the patient as a consumer of the information. This will empower women with the possibility of obtaining the best information to make informed choices.
